# Disrupted dynamic amplitude of low-frequency fluctuations in patients with active thyroid-associated ophthalmopathy

**DOI:** 10.3389/fcell.2023.1174688

**Published:** 2023-05-12

**Authors:** Zhi Wen, Yan Kang, Yu Zhang, Huaguang Yang, Yilin Zhao, Xin Huang, Baojun Xie

**Affiliations:** ^1^ Department of Radiology, Renmin Hospital of Wuhan University, Wuhan, China; ^2^ State Key Laboratory of Magnetic Resonance and Atomic and Molecular Physics, National Center for Magnetic Resonance in Wuhan, Wuhan Institute of Physics and Mathematics, Innovation Academy for Precision Measurement Science and Technology, Chinese Academy of Sciences, Wuhan, China; ^3^ University of Chinese Academy of Sciences, Beijing, China; ^4^ Department of Ophthalmology, Jiangxi Provincial People’s Hospital, The First Affiliated Hospital of Nanchang Medical College, Nanchang, Jiangxi, China

**Keywords:** thyroid-associated ophthalmopathy, active phase, visual dysfunction, dynamic amplitude of low-frequency fluctuation, support vector machine

## Abstract

**Purpose:** Thyroid-associated ophthalmopathy (TAO) is an autoimmune disease that affects the orbit and is the most prevalent extra-thyroidal complication of Graves’ disease. Previous neuroimaging studies have focused on abnormal static regional activity and functional connectivity in patients with TAO. However, the characteristics of local brain activity over time are poorly understood. This study aimed to investigate alterations in the dynamic amplitude of low-frequency fluctuation (dALFF) in patients with active TAO and to distinguish patients with TAO from healthy controls (HCs) using a support vector machine (SVM) classifier.

**Methods:** A total of 21 patients with TAO and 21 HCs underwent resting-state functional magnetic resonance imaging scans. dALFFs were calculated in conjunction with sliding window approaches to assess dynamic regional brain activity and to compare the groups. Then, we used SVM, a machine learning algorithm, to determine whether dALFF maps may be used as diagnostic indicators for TAO.

**Results:** Compared with HCs, patients with active TAO showed decreased dALFF in the right calcarine, lingual gyrus, superior parietal lobule, and precuneus. The SVM model showed an accuracy of 45.24%–47.62% and area under the curve of 0.35–0.44 in distinguishing TAO from HCs. No correlation was found between clinical variables and regional dALFF.

**Conclusion:** Patients with active TAO showed altered dALFF in the visual cortex and the ventral and dorsal visual pathways, providing further details on the pathogenesis of TAO.

## 1 Introduction

Thyroid-associated ophthalmopathy (TAO), commonly known as Graves’ ophthalmopathy, is an autoimmune disease that affects the orbit and is the most prevalent extra-thyroidal complication of Graves’ disease. The main pathological changes of TAO were extraocular muscle swelling and periorbital fat increase. Although the pathogenesis is not fully understood, the thyroid-stimulating hormone receptor (TSHR) is considered the main target of the autoimmune reaction (Hiromatsu et al., 2014). The activation of autoantibodies against TSHR mediates inhibiting, interfering with, or stimulating intracellular signal transduction. In patients with TAO, an over-expression of TSHR is observed in the thyroid, extraocular muscles, and retrobulbar tissue, particularly in orbital fibroblasts, leading to extraocular muscle swelling, expansion of the orbital adipose tissue, and high intraocular pressure. Long-lasting orbital edema causes the extraocular muscles to fibrose and/or atrophy, which results in restrictive strabismus (Weiler, 2017). The swelling of extraocular muscles directly compresses the optic nerve at the orbital apex. The orbital fat expands, resulting in overt exophthalmos, which may lead to stretching and injury of the optic nerve. Compression and circulatory obstruction of optic nerve fibers may lead to denervated atrophy (Bartalena et al., 2021; Song et al., 2022).

The course of TAO includes an active and an inactive phase (Weiler, 2017). In the active stage, TAO has severe inflammatory lymphocyte infiltration, edema, and fibroblast proliferation in orbital tissues (Hiromatsu et al., 2014) and presents typical eye symptoms, including exophthalmos and diplopia, which can lead to retinal damage, optic neuropathy, and even blindness in severe cases (Bartalena et al., 2021). Active immunosuppression intervention may be helpful to limit the destructive and fibrotic consequences of the immune cascade and release aggravation of eye symptoms. In the chronic stage, orbital residual fibrosis persists, and there is little response to medical treatment that requires surgery. Therefore, understanding the pathological mechanism of active TAO is helpful to save vision at an early stage.

Neuroimaging studies demonstrated that the abnormal brain structural and functional changes were associated with the visual and cognitive impairments in TAO. For example, diffusion tension imaging studies showed significantly decreased fractional anisotropy (FA) in the optic nerve in TAO that correlated negatively with visual field defects and positively with clinical activity scores (CAS) (Ozkan et al., 2015), “NO SPECS” classification, and extraocular muscle thickness (Lee et al., 2018). Compared with healthy controls (HCs), patients with active TAO had cortical thinning in the left lateral occipital sulcus, left fusiform gyrus, right precuneus, right superior frontal cingulate, right superior periparietal gyrus, right paracentral gyrus, right postcentral gyrus, and right insula (Silkiss and Wade, 2016) but increased gray matter volume (GMV) in the right inferior frontal gyrus, left superior frontal gyrus (SFG), left orbital SFG, left orbital middle frontal gyrus, left precuneus, and left postcentral gyrus (Luo et al., 2022). Resting-state functional MRI (rs-fMRI) studies demonstrated decreased amplitudes of low-frequency fluctuations (ALFFs) in the left middle occipital gyrus (MOG), superior occipital gyrus, and precuneus (Chen et al., 2021c), decreased fractional ALFF (fALFF) in the right calcarine, and increased fALFF in the right inferior temporal gyrus and left posterior cingulate cortex in active TAO relative to HCs (Zhu et al., 2022). The microvascular density of the optic nerve head was negatively correlated with fALFF in the right calcarine, while it was positively correlated with fALFF in the posterior cingulate cortex (Zhu et al., 2022). Another study found decreased regional homogeneity in the right MOG and the right angular gyrus, reduced ALFF in the right superior occipital gyrus and bilateral precuneus, decreased voxel-mirrored homotopic connectivity (VMHC) between calcarine, angular gyri, and MOG, and decreased functional connectivity between the calcarine/lingual gyri and the contralateral middle temporal gyrus (MTG) (Qi et al., 2021, 2022; Wen et al., 2022). There are a few neuroimaging results focused on the neural activity differences between active and inactive TAO. A direct comparison of the active and inactive phases of TAO found increased GMV in the right MTG, left SFG, and left precuneus (Luo et al., 2022), as well as increased ALFF in bilateral precuneus (Chen et al., 2021c). ALFF values in the bilateral precuneus were positively correlated with CAS and mini-mental state examination (MMSE) scores and negatively correlated with disease duration (Chen et al., 2021c). Moreover, in the hyperthyroid condition, gray matter volumes were increased in the right cerebellum lobule VI and decreased in the bilateral visual cortex and cerebellum lobules I–IV, the ALFF was decreased in the right posterior cingulate cortex, and the FC was increased in the bilateral anterior insula, posterior insula, and cerebellum anterior lobe, compared to the euthyroid condition, again suggesting a crucial role for the cerebellum in the mediation of TH effects (Gobel et al., 2020). Thus, these studies have demonstrated that patients with active TAO may provide evidence of the pathological mechanisms underlying active TAO.

fMRI can non-invasively measure neuronal activity, deepening our understanding of visual processing and perception. Previous studies assumed that the BOLD signal was static during the fMRI scanning. However, recent research has proposed that neural activity is dynamic over time (Liu and Duyn, 2013). Evidence from task-based fMRI and electrophysiology research showed that functional activities and connectivity may show dynamic changes in the time scale of several seconds to several minutes (Liegeois et al., 2017). Compared with static analysis by using the average functional activity or connectivity, dynamic analysis is helpful to observe the details in static analysis and can provide a deeper understanding of the basic mechanism of brain activity and connectivity. The ALFF method calculates the power within the effective frequency range (0.01–0.08 Hz) of each voxel in the brain and reflects the spontaneous activity of neurons at rest (Biswal et al., 2010). Dynamic ALFF (dALFF) is an extension of ALFF, which studies the temporal variability of brain activity and provides information on the changes in ALFF with time by combining the sliding window method. The decrease in dALFF represents functional impairment, while the increase in dALFF represents unstable neural activity. The support vector machine (SVM) model is a focused area of machine learning in recent years and shows its advantages in small samples, especially when the sample size is far less than the feature dimension. Combining dALFF and SVM classification analysis has been applied in various disease conditions, such as comitant exotropia (Chen et al., 2022a), transient ischemic attack (Ma et al., 2021), and Parkinson’s disease (Zhang et al., 2019), and showed good performance to distinguish patients and healthy controls.

Our hypotheses show that the dynamic changes in spontaneous brain activity may present a disease-related pattern in patients with TAO. In the present study, we aimed to investigate dynamic alterations in local resting-state metrics and to explore whether dALFF could be used as a diagnostic tool for TAO.

## 2 Methods

### 2.1 Ethics approval

This cross-sectional study was approved by the Research Ethics Committee of Jiangxi Provincial People’s Hospital and adhered to the guidelines of the Declaration of Helsinki. All patients with TAO and HCs provided written informed consent before participation.

### 2.2 Participants

The pool of patients with TAO and HCs was identical to that reported in previous studies (Qi et al., 2021, 2022; Wen et al., 2022). A total of 21 patients with TAO (seven females’ mean age, 54.17 ± 4.83 years) were enrolled from the Departments of Ophthalmology and Endocrinology, Jiangxi Provincial People’s Hospital.

Patients in the active phase of TAO who were right-handed were included in the TAO group. The diagnosis of TAO was made by two experienced ophthalmologists according to the diagnostic criteria for Graves’ ophthalmopathy (Bartley and Gorman, 1995), measuring visual acuity, visual field, color vision, and the pupil reflex. The disease activity of TAO was evaluated according to the modified 7-point Mourits’ CAS, and the active phase was defined by a CAS equal to or greater than 3 (Bartalena et al., 2021). The exclusion criteria for the TAO and HC groups were as follows: (1) severe TAO with dysthyroid optic neuropathy; (2) symptoms caused by other ocular diseases, such as glaucoma, vitreous hemorrhage, high myopia, strabismus, cataract, and optic neuritis; (2) history of eye trauma or surgery; (3) history of neurological and psychiatric disorders; (4) alcohol or drug abuse; and (5) contraindications to MRI, such as claustrophobia or implanted pacemakers.

A total of 21 HCs (seven females’ mean age: 55.17 ± 5.37 years) matched for age, sex, handedness, and educational level were also recruited. HCs were psychologically healthy, with no history of TAO, and had normal or corrected-to-normal vision. The exclusion criteria for the TAO and HC groups were as follows: (1) symptoms caused by other ocular diseases, such as glaucoma, vitreous hemorrhage, high myopia, and strabismus; (2) history of eye trauma or surgery; (3) history of neurological and psychiatric disorders; (4) alcohol or drug abuse; and (5) contraindications to MRI, such as claustrophobia or implanted pacemakers.

### 2.3 Clinical assessment

All patients underwent comprehensive eye examinations that included measuring intraocular pressure (IOP), eyeball protrusion, best-corrected visual acuity (BCVA), and performing slit lamp examinations and retinal fundoscopy. In addition, the TAO duration was confirmed by self-reports from patients and was determined as the interval between the onset of TAO-related clinical symptoms and the date of the MRI examination.

### 2.4 MRI data acquisition

MRI scanning was performed on a 3-T MR scanner (Discovery 750W System; GE Healthcare, Chicago, IL, United States) with an 8-channel head coil. Foam pads were placed on both sides of the jaw to limit head movements, and earplugs were used to attenuate noise during scanning. During data acquisition, all participants were asked to close their eyes, stay awake, and not to think about anything in particular.

High-resolution T1-weighted images covering the whole brain were acquired with a magnetization-prepared rapid gradient echo (MPRAGE) sequence, with the following parameters: repetition time (TR) = 8.5 ms, echo time (TE) = 3.3 ms, flip angle = 12°, slice thickness = 1.0 mm, slice gap = 0 mm, voxel size = 1 × 1 × 1 mm^3^, field of view (FOV) = 240 × 240 mm^2^, matrix size = 256 × 256, and sagittal slice number = 176. Rs-fMRI involved a gradient-recalled echo (GRE) planar imaging sequence, with the following parameters: TR = 2000 ms, TE = 25 ms, flip angle = 90°, FOV = 240 × 240 mm^2^, matrix size = 64 × 64, voxel size = 3.6 × 3.6 × 3.6 mm^3^, axial slice number = 35, and volume numbers = 240. T2-weighted imaging and T2 fluid-attenuated inversion recovery images were acquired to exclude brain lesions. The total scanning time for each subject was 15 min.

### 2.5 fMRI data preprocessing

rs-fMRI data were preprocessed using the toolbox for Data Processing & Analysis of Brain Imaging (DPABI; http://www.rfmri.org/dpabi) and Statistical Parametric Mapping 8 (SPM8, http://www.fil.ion.ucl.ac.uk) implemented in MATLAB (2013a; MathWorks, Natick, MA, United States). Preprocessing included the following steps: (1) Original DICOM-format files were converted into the NIfTI format. (2) The first 10 time points for each subject were removed due to the signal reaching equilibrium and the participants adapting to scanning noise. (3) Slice-timing and motion correction were performed; subjects with a maximum displacement of less than 1.5 mm in any cardinal direction (x, y, z) and a maximum spin (x, y, z) of less than 1.5 were included in the following analysis. (4) Each T1 image was co-registered to the mean functional image and was segmented into gray matter, white matter, and cerebrospinal fluid using the Diffeomorphic Anatomical Registration Through Exponentiated Lie Algebra approach (Ashburner, 2007). (5) Functional images were normalized to the Montreal Neurological Institute space, resampled with a voxel size of 3 × 3 × 3 mm, and smoothed with a Gaussian kernel with a full-width at half maximum of 6 mm. This was followed by (6) detrending and (7) applying a temporal filter (0.01–0.08 Hz) to reduce the influence of low-frequency drift and high-frequency noise. Subsequently, the images were used to compute the dALFF maps.

### 2.6 Calculation of dALFF

To obtain ALFF values, the time series for each voxel were transformed to the frequency domain using a fast Fourier transform, and the power spectrum was then obtained using DPABI software. The square root of the power spectrum was z-transformed using Fisher’s r-to-z transformation to reduce the global effects of variability across participants.

The sliding window method was applied to evaluate the dALFF for each participant using the Temporal Dynamic Analysis (TDA) toolkit in DPABI software. The sliding window length affected the dALFF: the minimum window length should be larger than 1/fmin, where fmin is the minimum frequency of time series (Leonardi and Van De Ville, 2015). Liao et al. (2019) also found that when the step size of the sliding window is fixed, the variance of dALFF decreases with the increase in the sliding window length; when the window length is kept at 50 TRs, the variance of dALFF is constant with the increase in the step size. Previous studies have shown that a window length of 50 TRs (100 s) is the optimal parameter to keep a balance between capturing rapidly shifting dynamic activity and achieving reliable brain activity estimation. Therefore, we chose 50 TRs as the sliding window length and 1 TRs as the step size to calculate the dALFF of each participant, which is in accordance with the methods of Liu et al. (2021) and Cui et al. (2020). The ALFF map was computed within each window, generating a set of ALFF maps for each participant. The standard deviation (SD) divided by the global mean value of the ALFF at each voxel across each window was calculated to assess the temporal variability of the ALFF, which is defined as dALFF.

### 2.7 Validation analysis

To verify our findings on the dALFF variability obtained using a sliding window length of 50 times the TR, we performed auxiliary analyses with different sliding window lengths. We recalculated the main results using window lengths of 30 and 80 times the TR, which is in accordance with the methods of Liu et al. (2021) and Cui et al. (2020).

### 2.8 Support vector machine analysis

We performed machine learning analyses using the SVM algorithm to determine whether dALFF maps can be used as potential diagnostic indicators of TAO. Using the Pattern Recognition for Neuroimaging Toolbox (Schrouff et al., 2013), the dALFF values of brain regions that differed between the groups were used as classification features. Then, leave-one-out cross-validation (LOOCV) was used to validate the SVM classifier. The accuracy, sensitivity, and specificity were used to quantify the performance of classification methods. The receiver operating characteristic curves and the corresponding areas under the curve (AUCs) were generated to assess the classification efficiency.

### 2.9 Statistical analysis

SPSS software (v22.0; IBM Corp., Armonk, NY, United States) was used to analyze clinical variables. *p*-values <0.05 were considered statistically significant. One-sample *t*-tests were performed in the Statistical Parametric Mapping software to assess the intragroup z-values of dALFF maps. Then, with age, sex, and total intracranial volume as covariates, the individual z-maps were entered into a two-sample *t*-test to identify differences between groups. The Gaussian random field method (Worsley et al., 1996) was used to correct for multiple comparisons, with a cluster-level *p* < 0.05 as statistically significant, corresponding to a two-tailed voxel level of *p* < 0.01.

### 2.10 Correlation analysis

Pearson correlation analysis was applied to determine the relationship between mean dALFF values and clinical factors in TAO, such as the severity of the disease, BCVA, and IOP. *p*-values below 0.05 were considered statistically significant.

## 3 Results

### 3.1 Demographics and disease characteristics

Sex, age, and educational level did not significantly differ between the TAO and HC groups (*p* = 1, *p* = 0.75, and *p* = 0.86, respectively). In comparison with HCs, the TAO group showed significantly worse BCVA (*p* < 0.05) and higher IOP (*p* < 0.001) in both eyes. Table 1 lists demographic and disease characteristics of the study sample.

**TABLE 1:** Characteristics of participants in TAO and HC groups.

Condition	TAO group	HC group	t	*p*
Gender (male/female)	14/7	14/7	N/A	1
Age (years)	54.17 ± 4.83	55.17 ± 5.37	−0.348	0.75
Duration (months)	11.25 ± 4.42	-	-	-
Education	11.17 ± 2.64	11.42 ± 1.95	−0.269	0.86
BCVA-OD	0.67 ± 0.35	1.14 ± 0.15	−4.462	0.026*
BCVA-OS	0.64 ± 0.29	1.06 ± 0.23	−4.297	0.023*
IOP-OD	25.81 ± 2.35	15.33 ± 1.20	−5.554	<0.001*
IOP-OS	25.62 ± 2.32	15.10 ± 1.11	−5.560	<0.001*

Notes: Independent sample *t*-test for the normally distributed continuous data (means ± SD). Chi-squared test for sex. *p < 0.05 indicated statistically significant. TAO, thyroid-associated ophthalmopathy; HC, healthy control; BCVA, best-corrected visual acuity; OD, oculus dexter; OS, oculus sinister; IOP, intraocular pressure.

### 3.2 dALFF in TAO and HC groups

**FIGURE 1:**
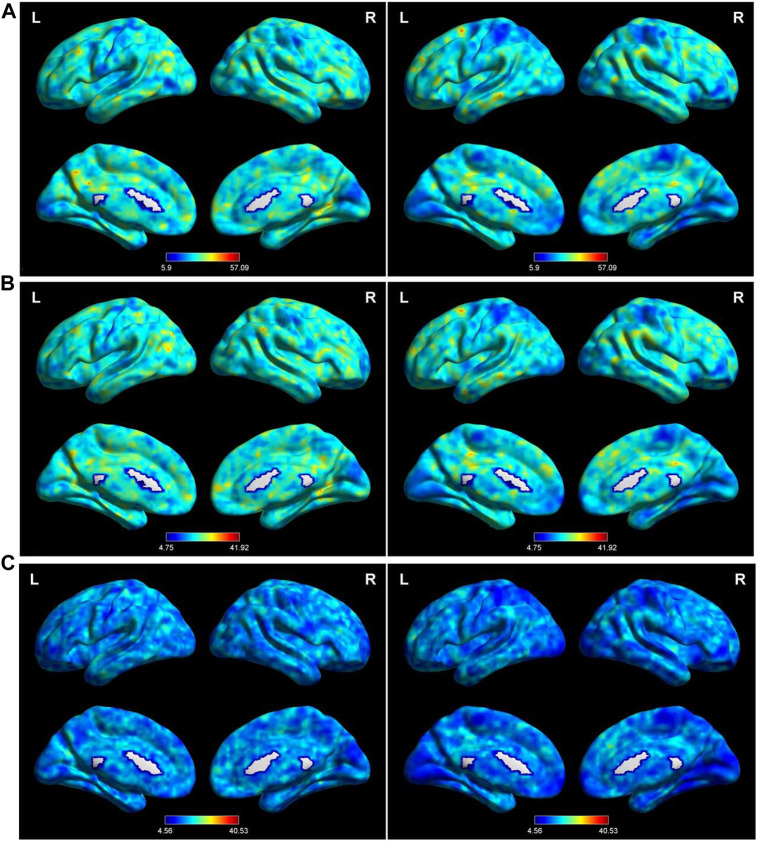
Distribution of dALFF using the following three distinct sliding window parameter settings in the typical frequency band (0.01–0.08 Hz) in TAO (left column) and HC (right column) groups: **(A)** window length of 30 TRs (60 s), **(B)** window length of 50 TRs (100 s), and **(C)** window length of 80 TRs (160 s). dALFF, dynamic amplitude of low-frequency fluctuation; TAO, thyroid-associated ophthalmopathy; HC, healthy control; L, left; R, right.

Setting the window length to 50 TR and the sliding step to 1 TR produced the main findings. Then, these were validated using different sliding window lengths. The spatial distributions of mean dALFF in the TAO and HC groups with window lengths of 30×, 50×, and 80× TR are shown in Figure 1.

**FIGURE 2:**
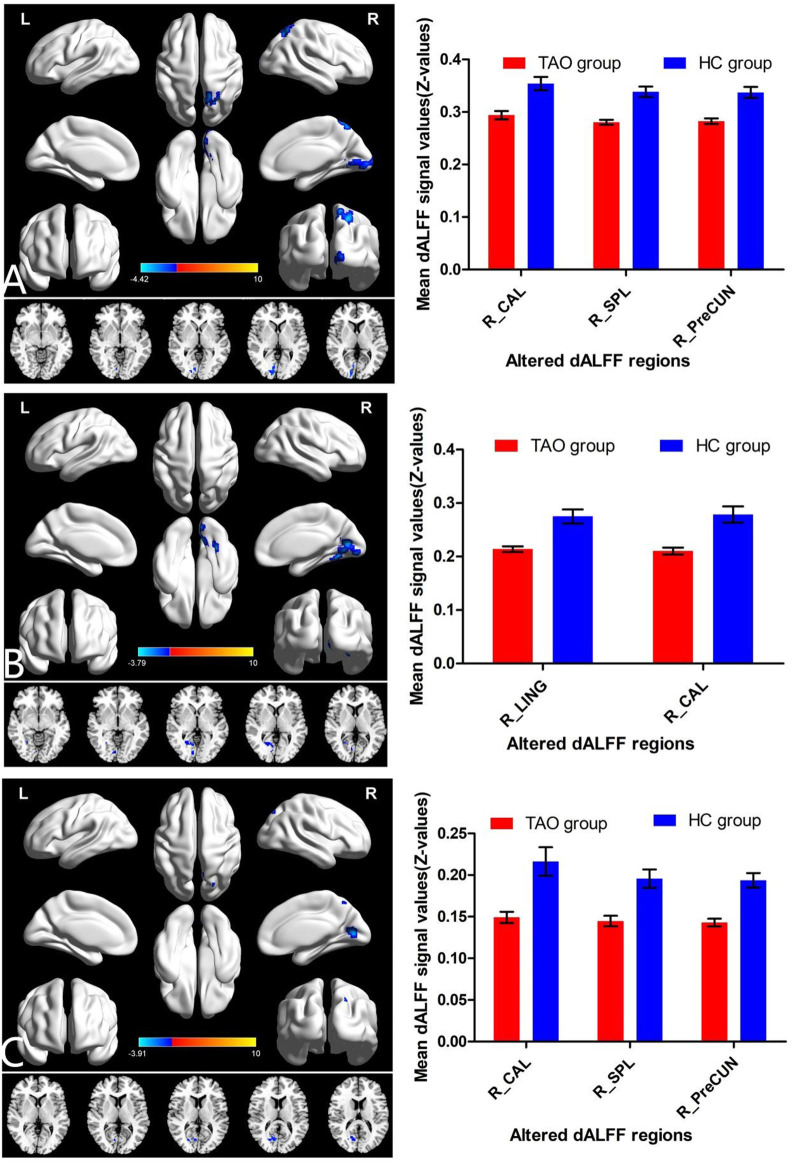
Brain regions with significant differences in dALFF between TAO and HC groups using the following three distinct sliding window parameter settings: **(A)** window length of 30 TRs (60 s), **(B)** window length of 50 TRs (100 s), and **(C)** window length of 80 TRs (160 s). The histogram shows the mean and standard deviation of dALFF values in these regions in TAO and HC groups. dALFF, dynamic amplitude of low-frequency fluctuation; TAO, thyroid-associated ophthalmopathy; HC, healthy control; LING, lingual gyrus; CAL, calcarine; PreCun, precuneus; SPL, superior parietal lobule; TR, time of repetition; L, left; R, right.

With a window length of ×50 TR and a sliding step of 1 TR, patients with TAO had significantly lower dALFF in two locations in the right hemisphere (calcarine gyrus [Brodmann’s area (BA) 17,18] and lingual gyrus [BA 18]), compared with HCs (Table 2, Figure 2B). Similarly, with window lengths of ×30 and ×80 TR, patients with TAO showed significantly lower dALFF in three locations in the right hemisphere (calcarine gyrus [BA 17,18], precuneus [BA 7], and superior parietal lobule (SPL; BA 7]) compared with HCs (Table 2, Figure 2A, Figure 2C).

**TABLE 2:** Brian regions with significant differences in dALFF values between TAO and HC groups (voxel-level *p* < 0.01 for Gaussian random field correction, cluster-level *p* < 0.05).

Region	Brodmann’s areas	Side	MNI coordinate			Peak T	Cluster size
			x	y	z		
TAO < HC, with a window size of 30 TRs and sliding step of 1 TR							
Calcarine	17, 18	R	21	−87	0	−3.30	40
Superior parietal lobule	7	R	30	−57	60	−4.42	47
Precuneus	7	R	9	−66	63	−3.60	40
TAO < HC, with a window size of 50 TRs and sliding step of 1 TR							
Calcarine	17, 18	R	21	−75	12	−3.79	50
Lingual gyrus	18	R	34	−60	0	−3.41	40
TAO < HC, with a window size of 80 TRs and sliding step of 1 TR							
Calcarine	17, 18	R	21	−75	12	−3.63	40
Superior parietal lobule	7	R	27	−81	51	−3.40	11
Precuneus	7	R	6	−72	57	−3.91	36

dALFF, dynamic amplitude of low-frequency fluctuation; TAO, thyroid-associated ophthalmopathy; HC, healthy control; MNI, Montreal Neurological Institute; GFR, Gaussian random field theory; TR, time of repetition; R, right.

### 3.3 Support vector machine classification

**FIGURE 3:**
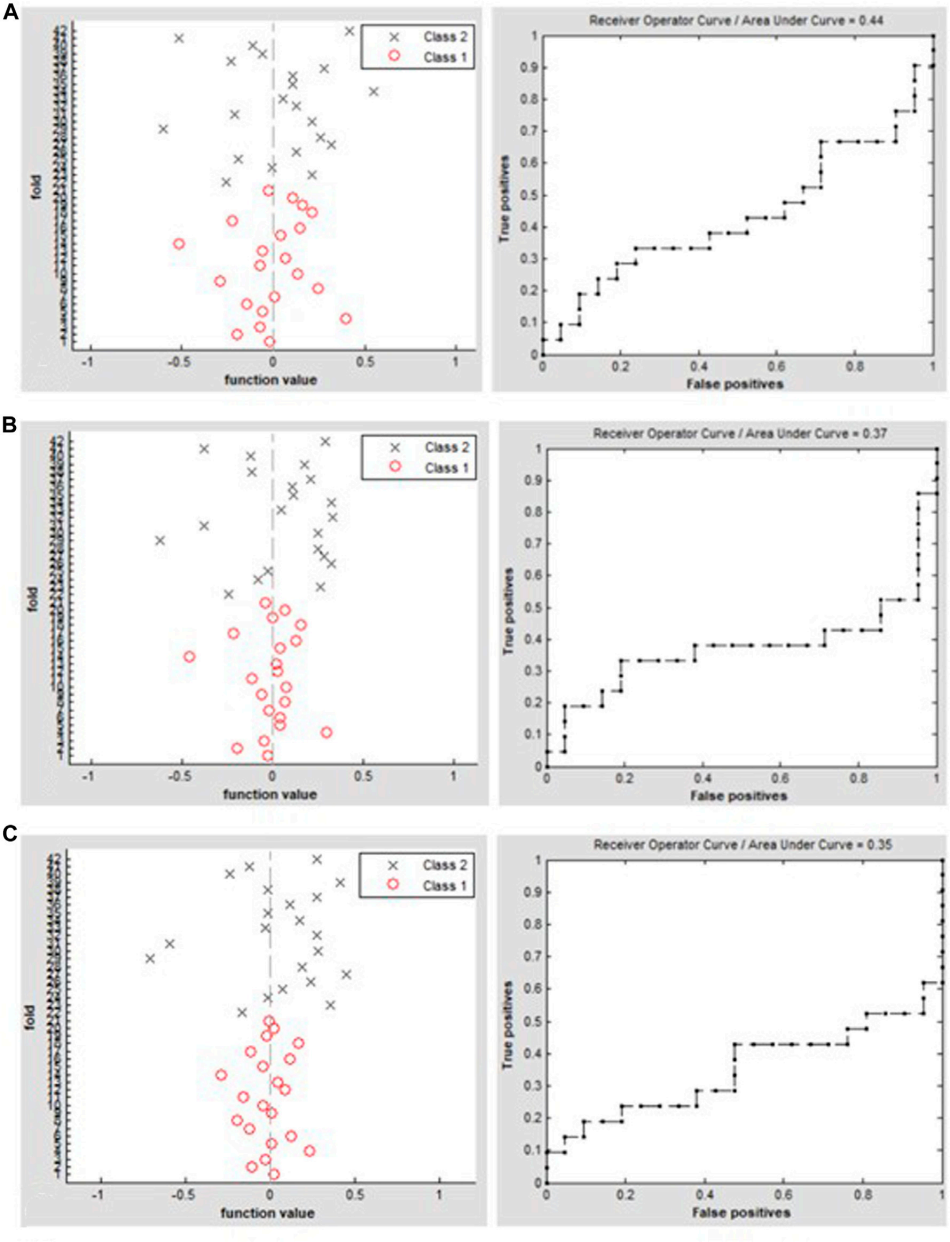
Classification results using SVM based on dALFF values using the following three distinct sliding window parameter settings: **(A)** window length of 30 TRs (60 s), **(B)** window length of 50 TRs (100 s), and **(C)** window length of 80 TRs (160 s). The left column of images shows a 10-fold in the class 1 (TAO group) and class 2 (HC group), and the right column shows the ROC curve of the SVM classifier with AUC values of 0.44, 0.37, and 0.35, respectively. dALFF, dynamic amplitude of low-frequency fluctuation; TAO, thyroid-associated ophthalmopathy; HC, healthy control; ROC, receiver operating characteristic; SVM, support vector machine; AUC, area under the curve.

Setting the window length to ×30, ×50, or ×80 TR with a sliding step of 1 TR, the SVM classification of dALFF achieved overall accuracies of 45.24%, 47.62%, and 45.24% and AUCs of 0.44, 0.37, and 0.35, respectively, for distinguishing between patients with TAO and HCs (Figure 3).

### 3.4 Correlation analysis

There was no correlation between mean dALFF values and disease duration, BCVA, or IOP in patients with TAO.

## 4 Discussion

This study used dALFF analysis to investigate the temporal variability in local brain activity in patients with TAO. Compared with HCs, patients with TAO exhibited decreased dALFF in several posterior brain regions in the right hemisphere such as the calcarine gyrus (BA 17,18) and lingual gyrus (BA 18). We used other sliding window lengths to verify our findings and found that the temporal variability of dALFF in the right calcarine was highly reproducible across different window lengths. In addition, dALFF in the right precuneus (BA 7) and right SPL (BA 7) in TAO was reduced by using window lengths equal to 30 and 80 TRs. Unfortunately, the dALFF variability in these regions could not be used to classify patients with TAO and HCs, achieving an accuracy of 45.24%–47.62% and AUC of 0.35–0.44, because AUCs of 0.5–0.7 indicate low accuracy and 0.7–0.9 represents higher accuracy. Indeed, no correlation was found between the clinical variables and the dALFF of these brain regions. Nonetheless, the present study emphasized the significance of considering dynamic local brain activity in TAO.

We found that the spatial pattern of temporal variability of dALFF in the right calcarine was highly reproducible across different window lengths. The calcarine is regarded as the part of the primary visual cortex (V1). On the one hand, it receives visual inputs from the retina via thalamic relays and is associated with visual field defects and blurred vision. On the other hand, it receives visual information from the lateral geniculate body and is the core component of binocular vision, creating depth perception (Weiler, 2017). Dysfunction in the primary visual cortex is a common finding among various eye diseases such as glaucoma (Chen et al., 2022b), diabetic retinopathy (Huang et al., 2021), exotropia and amblyopia (Liang et al., 2016; Chen J. et al., 2021), and optic neuropathy (Sujanthan et al., 2022). Compared with HCs, patients with active TAO demonstrated decreased fALFF in the right calcarine (Zhu et al., 2022) and decreased FC between hemispheric calcarine gyri (Qi et al., 2022; Wen et al., 2022). The microvascular density of the optic nerve head has been negatively correlated with fALFF in the right calcarine (Zhu et al., 2022). Patients with TAO and in the euthyroid status showed decreased fALFF in the bilateral calcarine (Chen et al., 2021d) and decreased VMHC in lingual gyri/calcarine (Chen et al., 2021b). The VMHC of the lingual gyri/calcarine was positively correlated with visual acuity (Chen et al., 2021b). Moreover, patients with hyperthyroidism and without orbital symptoms showed reduced GMV in the bilateral calcarine, suggesting a preclinical stage of TAO (Zhang et al., 2014). However, no particular differences were observed in the calcarine gyrus in patients with inactive TAO, relative to HCs or patients with active TAO (Chen et al., 2021c; Luo et al., 2022). In the present study, patients with active TAO consistently demonstrated decreased dALFF variability in the right calcarine (BA 17, 18); findings obtained from three different sliding window values of ×30, ×50, and ×80 TRs were in line with the aforementioned studies. Altogether, static and dynamic dysfunctions of the calcarine might be promising indicators in TAO, which may be associated with impaired retinal projection and binocular fusion.

The visual pathway consists of ventral and dorsal streams. The ventral stream originates from V1 and projects to the inferior temporal cortex. The lingual gyrus is a key hub in the ventral stream and is commonly known as the ventral occipitotemporal region (lingual, fusiform, and parahippocampal gyri) and participates in face recognition (Dinkelacker et al., 2011). In the present study, dALFF was lower in the right lingual gyrus in active TAO patients than that in HCs, which is consistent with the previous finding of reduced FC between the contralateral MTG and bilateral calcarine/lingual gyri (Wen et al., 2022). This suggested that the ventral stream, which processes faces, was impaired (Reisch et al., 2022).

The dorsal visual stream starts from V1 and projects to the posterior parietal cortex, which is important for binocular vision fusion, and has predominant advantages in decoding the disparities present in 3D images. The SPL forms the association cortex of the parietal lobe and is responsible for visual-motor coordination (Wolpert et al., 1998). Located medial to the SPL, the precuneus is a component of the dorsal stream as well as the default mode network. It receives visual information from cortical area V5, which plays an important role in visuospatial imagination and plot memory extraction (Cavanna and Trimble, 2006). Most previous imaging studies have shown that the structure and function of the precuneus changed in TAO. However, there have been inconsistencies in the results. Compared with HCs, the right precuneus of patients with active TAO was atrophied, which indicated cognitive impairment (Silkiss and Wade, 2016). In contrast, compared with HCs and patients with inactive TAO, the GMV of the left precuneus of patients with active TAO was increased. The GMV of the right precuneus was positively correlated with CAS, left exophthalmos, and quality of life in thyroid eye disease (TED-QOL), while it was negatively correlated with right eye visual acuity (Luo et al., 2022). Qi et al. (2021) found that ALFF of the bilateral precuneus was lower in patients with active TAO, while Chen et al. (2021c) did not find differences between patients with active TAO and HCs. The ALFF in the right precuneus in TAO was positively correlated with CAS and MMSE scores but negatively correlated with disease duration (Chen et al., 2021c). Our study found decreased dALFF in the right precuneus and speculated that it was related to the slow processing speed of visual spatial information. In future studies, more attention needs to be paid to the importance of the precuneus in TAO.

In the present study, the SVM classification was adopted, and dALFF was used as a feature to distinguish patients with active TAO from HCs. Unfortunately, the dALFF variability in these regions only achieved an accuracy of 45.24%–47.62% and AUCs of 0.35–0.44, indicating poor accuracy. Hence, which indicator could be most sensitive to detecting TAO-related brain changes has to be determined yet.

There were some limitations to the present study. First, the sample size was small. Second, this study recruited patients with active TAO to explore TAO-specific brain functional changes without controlling for levels of thyroid hormones. Changes in thyroid hormone have short- and long-term effects on brain function (Gobel et al., 2020). Other studies that recruited patients with TAO and in a hematologically euthyroid state (Chen et al., 2021d; Jiang et al., 2022; Zhou et al., 2022), or those that performed longitudinal monitoring of thyroid hormone levels, may provide additional evidence for understanding the role of thyroid hormones in the visual and cognitive impairments seen in TAO.

In conclusion, this study found that in the pathogenesis of TAO, the dALFF in visual cortex and ventral and dorsal pathways decreased. This might indicate that patients with TAO may need to consider neuroprotective therapy in the future.

## Data Availability

The raw data supporting the conclusion of this article will be made available by the authors, without undue reservation.

## References

[B1] Ashburner J. (2007). A fast diffeomorphic image registration algorithm. Neuroimage 38 (1), 95–113. 10.1016/j.neuroimage.2007.07.007 17761438

[B2] Bartalena L. Kahaly G. J. Baldeschi L. Dayan C. M. Eckstein A. Marcocci C. (2021). The 2021 European Group on Graves' orbitopathy (EUGOGO) clinical practice guidelines for the medical management of Graves' orbitopathy. Eur. J. Endocrinol. 185 (4), G43–G67. 10.1530/EJE-21-0479 34297684

[B3] Bartley G. B. Gorman C. A. (1995). Diagnostic criteria for Graves' ophthalmopathy. Am. J. Ophthalmol. 119 (6), 792–795. 10.1016/s0002-9394(14)72787-4 7785696

[B4] Biswal B. B. Mennes M. Zuo X. N. Gohel S. Kelly C. Smith S. M. (2010). Toward discovery science of human brain function. Proc. Natl. Acad. Sci. U. S. A. 107 (10), 4734–4739. 10.1073/pnas.0911855107 20176931 PMC2842060

[B5] Cavanna A. E. Trimble M. R. (2006). The precuneus: A review of its functional anatomy and behavioural correlates. Brain 129 (3), 564–583. 10.1093/brain/awl004 16399806

[B6] Chen J. Jin H. Zhong Y. L. Huang X. (2021a). Abnormal low-frequency oscillations reflect abnormal eye movement and stereovision in patients with comitant exotropia. Front. Hum. Neurosci. 15, 754234. 10.3389/fnhum.2021.754234 34690728 PMC8531266

[B7] Chen R. B. Ye S. Y. Pei C. G. Zhong Y. L. (2022a). Altered temporal dynamics of the amplitude of low-frequency fluctuations in comitant exotropia patients. Front. Hum. Neurosci. 16, 944100. 10.3389/fnhum.2022.944100 35911599 PMC9326226

[B8] Chen R. B. Zhong Y. L. Liu H. Huang X. (2022b). Machine learning analysis reveals abnormal functional network hubs in the primary angle-closure glaucoma patients. Front. Hum. Neurosci. 16, 935213. 10.3389/fnhum.2022.935213 36092649 PMC9450012

[B9] Chen W. Hu H. Wu Q. Chen L. Zhou J. Chen H. H. (2021b). Altered static and dynamic interhemispheric resting-state functional connectivity in patients with thyroid-associated ophthalmopathy. Front. Neurosci. 15, 799916. 10.3389/fnins.2021.799916 34938158 PMC8685321

[B10] Chen W. Wu Q. Chen L. Zhou J. Chen H. H. Xu X. Q. (2021d). Aberrant brain voxel-wise resting state fMRI in patients with thyroid-associated ophthalmopathy. J. Neuroimaging 31 (4), 773–783. 10.1111/jon.12858 33817897

[B11] Chen W. Wu Q. Chen L. Zhou J. Chen H. H. Xu X. Q. (2021c). Disrupted spontaneous neural activity in patients with thyroid-associated ophthalmopathy: A resting-state fMRI study using amplitude of low-frequency fluctuation. Front. Hum. Neurosci. 15, 676967. 10.3389/fnhum.2021.676967 34177495 PMC8226248

[B12] Cui Q. Sheng W. Chen Y. Pang Y. Lu F. Tang Q. (2020). Dynamic changes of amplitude of low-frequency fluctuations in patients with generalized anxiety disorder. Hum. Brain Mapp. 41 (6), 1667–1676. 10.1002/hbm.24902 31849148 PMC7267950

[B13] Dinkelacker V. Gruter M. Klaver P. Gruter T. Specht K. Weis S. (2011). Congenital prosopagnosia: Multistage anatomical and functional deficits in face processing circuitry. J. Neurol. 258 (5), 770–782. 10.1007/s00415-010-5828-5 21120515 PMC3090571

[B14] Gobel A. Gottlich M. Reinwald J. Rogge B. Uter J. C. Heldmann M. (2020). The influence of thyroid hormones on brain structure and function in humans. Exp. Clin. Endocrinol. Diabetes 128 (6-07), 432–436. 10.1055/a-1101-9090 32040963

[B15] Hiromatsu Y. Eguchi H. Tani J. Kasaoka M. Teshima Y. (2014). Graves' ophthalmopathy: Epidemiology and natural history. Intern Med. 53 (5), 353–360. 10.2169/internalmedicine.53.1518 24583420

[B16] Huang X. Wen Z. Qi C. X. Tong Y. Shen Y. (2021). Dynamic changes of amplitude of low-frequency fluctuations in patients with diabetic retinopathy. Front. Neurol. 12, 611702. 10.3389/fneur.2021.611702 33643197 PMC7905082

[B17] Jiang W. H. Chen H. H. Chen W. Wu Q. Chen L. Zhou J. (2022). Altered long- and short-range functional connectivity density in patients with thyroid-associated ophthalmopathy: A resting-state fMRI study. Front. Neurol. 13, 902912. 10.3389/fneur.2022.902912 35812093 PMC9259934

[B18] Lee H. Lee Y. H. Suh S. I. Jeong E. K. Baek S. Seo H. S. (2018). Characterizing intraorbital optic nerve changes on diffusion tensor imaging in thyroid eye disease before dysthyroid optic neuropathy. J. Comput. Assist. Tomogr. 42 (2), 293–298. 10.1097/RCT.0000000000000680 28937496

[B19] Leonardi N. Van De Ville D. (2015). On spurious and real fluctuations of dynamic functional connectivity during rest. Neuroimage 104, 430–436. 10.1016/j.neuroimage.2014.09.007 25234118

[B20] Liang M. Xie B. Yang H. Yu L. Yin X. Wei L. (2016). Distinct patterns of spontaneous brain activity between children and adults with anisometropic amblyopia: A resting-state fMRI study. Graefes Arch. Clin. Exp. Ophthalmol. 254 (3), 569–576. 10.1007/s00417-015-3117-9 26245338

[B21] Liao W. Li J. Ji G. J. Wu G. R. Long Z. Xu Q. (2019). Endless fluctuations: Temporal dynamics of the amplitude of low frequency fluctuations. IEEE Trans. Med. Imaging 38 (11), 2523–2532. 10.1109/TMI.2019.2904555 30872224

[B22] Liegeois R. Laumann T. O. Snyder A. Z. Zhou J. Yeo B. T. T. (2017). Interpreting temporal fluctuations in resting-state functional connectivity MRI. Neuroimage 163, 437–455. 10.1016/j.neuroimage.2017.09.012 28916180

[B23] Liu J. Bu X. Hu X. Li H. Cao L. Gao Y. (2021). Temporal variability of regional intrinsic neural activity in drug-naive patients with obsessive-compulsive disorder. Hum. Brain Mapp. 42 (12), 3792–3803. 10.1002/hbm.25465 33949731 PMC8288087

[B24] Liu X. Duyn J. H. (2013). Time-varying functional network information extracted from brief instances of spontaneous brain activity. Proc. Natl. Acad. Sci. U. S. A. 110 (11), 4392–4397. 10.1073/pnas.1216856110 23440216 PMC3600481

[B25] Luo L. Wen H. Gao L. Li R. Wang S. Wang Z. (2022). Morphological brain changes between active and inactive phases of thyroid-associated ophthalmopathy: A voxel-based morphometry study. Brain Res. 1790, 147989. 10.1016/j.brainres.2022.147989 35738426

[B26] Ma H. Huang G. Li M. Han Y. Sun J. Zhan L. (2021). The predictive value of dynamic intrinsic local metrics in transient ischemic attack. Front. Aging Neurosci. 13, 808094. 10.3389/fnagi.2021.808094 35221984 PMC8868122

[B27] Ozkan B. Anik Y. Katre B. Altintas O. Gencturk M. Yuksel N. (2015). Quantitative assessment of optic nerve with diffusion tensor imaging in patients with thyroid orbitopathy. Ophthalmic Plast. Reconstr. Surg. 31 (5), 391–395. 10.1097/IOP.0000000000000359 25549295

[B28] Qi C. X. Wen Z. Huang X. (2022). Reduction of interhemispheric homotopic connectivity in cognitive and visual information processing pathways in patients with thyroid-associated ophthalmopathy. Front. Hum. Neurosci. 16, 882114. 10.3389/fnhum.2022.882114 35865354 PMC9295451

[B29] Qi C. X. Wen Z. Huang X. (2021). Spontaneous brain activity alterations in thyroid-associated ophthalmopathy patients using amplitude of low-frequency fluctuation: A resting-state fMRI study. Neuroreport 32 (18), 1416–1422. 10.1097/WNR.0000000000001745 34776504

[B30] Reisch L. M. Wegrzyn M. Mielke M. Mehlmann A. Woermann F. G. Bien C. G. (2022). Face processing and efficient recognition of facial expressions are impaired following right but not left anteromedial temporal lobe resections: Behavioral and fMRI evidence. Neuropsychologia 174, 108335. 10.1016/j.neuropsychologia.2022.108335 35863496

[B31] Schrouff J. Rosa M. J. Rondina J. M. Marquand A. F. Chu C. Ashburner J. (2013). PRoNTo: Pattern recognition for neuroimaging toolbox. Neuroinformatics 11 (3), 319–337. 10.1007/s12021-013-9178-1 23417655 PMC3722452

[B32] Silkiss R. Z. Wade A. R. (2016). Neuroanatomic variations in Graves' dysthyroid ophthalmopathy as studied with MRI. Trans. Am. Ophthalmol. Soc. 114, T9. 28588345 PMC5444882

[B33] Song C. Luo Y. Yu G. Chen H. Shen J. (2022). Current insights of applying MRI in Graves' ophthalmopathy. Front. Endocrinol. (Lausanne) 13, 991588. 10.3389/fendo.2022.991588 36267571 PMC9577927

[B34] Sujanthan S. Shmuel A. Mendola J. D. (2022). Resting-state functional MRI of the visual system for characterization of optic neuropathy. Front. Hum. Neurosci. 16, 943618. 10.3389/fnhum.2022.943618 36330314 PMC9622755

[B35] Weiler D. L. (2017). Thyroid eye disease: A review. Clin. Exp. Optom. 100 (1), 20–25. 10.1111/cxo.12472 27701774

[B36] Wen Z. Wan X. Qi C. X. Huang X. (2022). Local-to-Remote brain functional connectivity in patients with thyroid-associated ophthalmopathy and assessment of its predictive value using machine learning. Int. J. Gen. Med. 15, 4273–4283. 10.2147/IJGM.S353649 35480997 PMC9037891

[B37] Wolpert D. M. Goodbody S. J. Husain M. (1998). Maintaining internal representations: The role of the human superior parietal lobe. Nat. Neurosci. 1 (6), 529–533. 10.1038/2245 10196553

[B38] Worsley K. J. Marrett S. Neelin P. Vandal A. C. Friston K. J. Evans A. C. (1996). A unified statistical approach for determining significant signals in images of cerebral activation. Hum. Brain Mapp. 4 (1), 58–73. 10.1002/(SICI)1097-0193(1996)4:1<58::AID-HBM4>3.0.CO;2-O 20408186

[B39] Zhang C. Dou B. Wang J. Xu K. Zhang H. Sami M. U. (2019). Dynamic alterations of spontaneous neural activity in Parkinson's disease: A resting-state fMRI study. Front. Neurol. 10, 1052. 10.3389/fneur.2019.01052 31632340 PMC6779791

[B40] Zhang W. Song L. Yin X. Zhang J. Liu C. Wang J. (2014). Grey matter abnormalities in untreated hyperthyroidism: A voxel-based morphometry study using the DARTEL approach. Eur. J. Radiol. 83 (1), e43–e48. 10.1016/j.ejrad.2013.09.019 24161779

[B41] Zhou J. Chen W. Wu Q. Chen L. Chen H. H. Liu H. (2022). Reduced cortical complexity in patients with thyroid-associated ophthalmopathy. Brain Imaging Behav. 16 (5), 2133–2140. 10.1007/s11682-022-00683-0 35821157

[B42] Zhu P. Liu Z. Lu Y. Wang Y. Zhang D. Zhao P. (2022). Alterations in spontaneous neuronal activity and microvascular density of the optic nerve head in active thyroid-associated ophthalmopathy. Front. Endocrinol. (Lausanne) 13, 895186. 10.3389/fendo.2022.895186 35937801 PMC9354054

